# Supplementary material for: Modeling glioblastoma heterogeneity as a dynamic network of cell states
Source: Mol Syst Biol. 2021 Sep 16;17(9):e10105. doi: 10.15252/msb.202010105 (PMC8444284; doi:10.15252/msb.202010105)
Supplement: Supplementary file 6 — Source Data for Figure 5 [file MSB-17-e10105-s004.zip › Figure5A_sourcedata/GSEA_3071/hallmarks_stateIII.GseaPreranked.1623742540963/HALLMARK_TNFA_SIGNALING_VIA_NFKB.html]

Details for gene set HALLMARK\_TNFA\_SIGNALING\_VIA\_NFKB[GSEA]

|  || Dataset | state33071 |
| Phenotype | NoPhenotypeAvailable |
| Upregulated in class | na\_neg |
| GeneSet | HALLMARK\_TNFA\_SIGNALING\_VIA\_NFKB |
| Enrichment Score (ES) | -0.3093105 |
| Normalized Enrichment Score (NES) | -1.7966444 |
| Nominal p-value | 0.0052083335 |
| FDR q-value | 0.024402188 |
| FWER p-Value | 0.29 |
Table: GSEA Results Summary

  

Fig 1: Enrichment plot: HALLMARK\_TNFA\_SIGNALING\_VIA\_NFKB      
 Profile of the Running ES Score & Positions of GeneSet Members on the Rank Ordered List

  

| PROBE | GENE SYMBOL | GENE\_TITLE | RANK IN GENE LIST | RANK METRIC SCORE | RUNNING ES | CORE ENRICHMENT || 1 | EIF1 |  |  | 86 | 0.426 | -0.0075 | No |
| 2 | HBEGF |  |  | 441 | -0.251 | -0.0905 | No |
| 3 | ATF3 |  |  | 515 | -0.255 | -0.1005 | No |
| 4 | REL |  |  | 530 | -0.256 | -0.0953 | No |
| 5 | ICAM1 |  |  | 568 | -0.258 | -0.0959 | No |
| 6 | LITAF |  |  | 582 | -0.259 | -0.0903 | No |
| 7 | PHLDA1 |  |  | 589 | -0.259 | -0.0828 | No |
| 8 | MYC |  |  | 599 | -0.260 | -0.0761 | No |
| 9 | MAFF |  |  | 735 | -0.266 | -0.1018 | No |
| 10 | RHOB |  |  | 865 | -0.272 | -0.1258 | No |
| 11 | DDX58 |  |  | 918 | -0.275 | -0.1297 | No |
| 12 | KLF4 |  |  | 973 | -0.278 | -0.1340 | No |
| 13 | ZC3H12A |  |  | 1024 | -0.281 | -0.1372 | No |
| 14 | SNN |  |  | 1196 | -0.290 | -0.1714 | No |
| 15 | BTG2 |  |  | 1350 | -0.297 | -0.2007 | No |
| 16 | CCNL1 |  |  | 1411 | -0.301 | -0.2058 | No |
| 17 | BIRC3 |  |  | 1469 | -0.304 | -0.2100 | No |
| 18 | TGIF1 |  |  | 1506 | -0.306 | -0.2087 | No |
| 19 | PFKFB3 |  |  | 1519 | -0.307 | -0.2011 | No |
| 20 | KLF6 |  |  | 1596 | -0.312 | -0.2100 | No |
| 21 | PTGER4 |  |  | 1628 | -0.314 | -0.2071 | No |
| 22 | CDKN1A |  |  | 1631 | -0.314 | -0.1967 | No |
| 23 | IFIH1 |  |  | 1695 | -0.318 | -0.2019 | No |
| 24 | DUSP4 |  |  | 1703 | -0.319 | -0.1927 | No |
| 25 | CD83 |  |  | 1729 | -0.321 | -0.1880 | No |
| 26 | ID2 |  |  | 1780 | -0.323 | -0.1897 | No |
| 27 | CEBPD |  |  | 1819 | -0.325 | -0.1882 | No |
| 28 | ACKR3 |  |  | 1865 | -0.328 | -0.1885 | No |
| 29 | PTPRE |  |  | 1880 | -0.328 | -0.1807 | No |
| 30 | MXD1 |  |  | 2053 | -0.338 | -0.2135 | No |
| 31 | SLC2A3 |  |  | 2117 | -0.343 | -0.2179 | No |
| 32 | PLAUR |  |  | 2181 | -0.347 | -0.2222 | No |
| 33 | RELB |  |  | 2243 | -0.352 | -0.2257 | No |
| 34 | IL7R |  |  | 2270 | -0.355 | -0.2202 | No |
| 35 | CD44 |  |  | 2471 | -0.371 | -0.2591 | No |
| 36 | BCL3 |  |  | 2598 | -0.382 | -0.2784 | No |
| 37 | DRAM1 |  |  | 2632 | -0.384 | -0.2736 | No |
| 38 | RNF19B |  |  | 2678 | -0.389 | -0.2718 | No |
| 39 | LAMB3 |  |  | 2757 | -0.395 | -0.2782 | No |
| 40 | PDE4B |  |  | 2878 | -0.406 | -0.2952 | Yes |
| 41 | LDLR |  |  | 2880 | -0.406 | -0.2813 | Yes |
| 42 | LIF |  |  | 2908 | -0.409 | -0.2741 | Yes |
| 43 | NINJ1 |  |  | 2923 | -0.411 | -0.2635 | Yes |
| 44 | JUN |  |  | 2935 | -0.413 | -0.2520 | Yes |
| 45 | EGR1 |  |  | 2961 | -0.415 | -0.2440 | Yes |
| 46 | BHLHE40 |  |  | 2981 | -0.417 | -0.2344 | Yes |
| 47 | TIPARP |  |  | 3072 | -0.428 | -0.2429 | Yes |
| 48 | TNFAIP3 |  |  | 3088 | -0.430 | -0.2318 | Yes |
| 49 | SMAD3 |  |  | 3091 | -0.430 | -0.2174 | Yes |
| 50 | TRIB1 |  |  | 3097 | -0.431 | -0.2037 | Yes |
| 51 | SGK1 |  |  | 3150 | -0.437 | -0.2020 | Yes |
| 52 | BMP2 |  |  | 3307 | -0.462 | -0.2264 | Yes |
| 53 | ABCA1 |  |  | 3421 | -0.485 | -0.2388 | Yes |
| 54 | JAG1 |  |  | 3424 | -0.486 | -0.2224 | Yes |
| 55 | PLAU |  |  | 3435 | -0.488 | -0.2080 | Yes |
| 56 | PLPP3 |  |  | 3439 | -0.490 | -0.1918 | Yes |
| 57 | SERPINE1 |  |  | 3474 | -0.499 | -0.1832 | Yes |
| 58 | ATP2B1 |  |  | 3580 | -0.539 | -0.1917 | Yes |
| 59 | FOS |  |  | 3621 | -0.561 | -0.1826 | Yes |
| 60 | CSF1 |  |  | 3669 | -0.590 | -0.1742 | Yes |
| 61 | G0S2 |  |  | 3728 | -0.637 | -0.1671 | Yes |
| 62 | PMEPA1 |  |  | 3737 | -0.657 | -0.1464 | Yes |
| 63 | F3 |  |  | 3764 | -0.693 | -0.1290 | Yes |
| 64 | TNFAIP6 |  |  | 3780 | -0.713 | -0.1081 | Yes |
| 65 | INHBA |  |  | 3801 | -0.746 | -0.0874 | Yes |
| 66 | EDN1 |  |  | 3838 | -0.812 | -0.0685 | Yes |
| 67 | FJX1 |  |  | 3843 | -0.821 | -0.0410 | Yes |
| 68 | PTX3 |  |  | 3858 | -0.855 | -0.0149 | Yes |
| 69 | TNC |  |  | 3874 | -0.951 | 0.0142 | Yes |
Table: GSEA details [plain text format]

  

Fig 2: HALLMARK\_TNFA\_SIGNALING\_VIA\_NFKB: Random ES distribution      
 Gene set null distribution of ES for **HALLMARK\_TNFA\_SIGNALING\_VIA\_NFKB**
